# Detection of anti-desmoglein antibodies in oral lichen planus: What do we know so far

**DOI:** 10.3389/fimmu.2022.1001970

**Published:** 2022-10-03

**Authors:** Dario Didona, Michael Hertl

**Affiliations:** Department of Dermatology and Allergology, Philipps University of Marburg, Marburg, Germany

**Keywords:** autoantibodies, desmoglein, epitope spreading, oral lichen planus, pemphigus vulgaris

## Abstract

Oral lichen planus (OLP) is an inflammatory disease of the oral mucosa. Clinically, two main subsets are described, namely non-erosive and erosive OLP. While non-erosive OLP is usually responsive to local therapies, erosive OLP is often refractory also to systemic therapies and extremely reduces the quality of life of the patients. Furthermore, in some erosive OLP cases different autoantibodies have been detected, including anti-desmoglein 1 and 3 autoantibodies, and anti-bullous pemphigoid 180 and 230 autoantibodies. However, their potential role is still not clear. In this paper, we reviewed the literature about the detection of autoantibodies against desmoglein 1 and 3, the main target antigens of pemphigus vulgaris, in patient with OLP, summarizing the more recent insights on this topic.

## Introduction

Oral lichen planus (OLP) is an inflammatory disease of the oral mucosa with a chronic course, that affects more often women in the fourth decade (1, 2). Classically, two main subtypes of OLP are described, namely non-erosive and erosive OLP (1, 2). The non-erosive type is clinically characterized by white streaks (Wickham´s striae), white plaques, and erythematous lesions, while the erosive type shows multiple painful erosions and ulcerations without well-defined borders and a necrotic base, that impair the quality of life of the patients (1, 2). Moreover, non-erosive OLP is usually asymptomatic and mostly responsive to topical therapies with corticosteroid or calcineurin inhibitors (3). By contrast, the therapy of erosive OLP is extremely tricky. Indeed, topical therapies are usually ineffective and patients need systemic therapies, which are mostly off-label, including methotrexate, hydroxychloroquine, and interleukin (IL) antagonists, such as guselkumab (anti-IL23 agent) or secukinumab (anti-IL 17 agent) (3, 4). In addition, in erosive OLP a malignant transformation has been reported in up to 5% of the patients (5). Several risk factors for malignant transformations have been proposed, including ulcerations of the tongue, female gender, and old age (5).

The pathogenesis of OLP is still unclear (6). The cellular-mediated immunity, triggered by several factors (e.g. genetic background, oral flora, and trauma), plays a pivotal role, resulting in the production of several proinflammatory molecules, including tumor necrosis factor (TNF)-alpha and interferon (IFN)-gamma (2). Indeed, the interaction between CD4+ T cells and CD8+ T cells, which causes the activation of cytotoxic activity by CD8+ T cells, represents the main factor in the pathogenesis of OLP (6). Furthermore, Th17/Tc17 cells and T cell-derived IL-17A have been described as important players in OLP, because they support the perpetuation of the inflammatory response in OLP, that is partially responsible for the chronic course of the disease (4).

Because of its clinical features, erosive OLP should be differentiated from pemphigus vulgaris (PV), a rare autoimmune disease that can affect both skin and oral mucosa (7). Indeed, the oral mucosa of PV patients shows painful ulcerations, which resemble the ones that clinically characterized erosive OLP. Furthermore, PV patients develop oral large erosions that usually impair massively the food intake (8). However, patients affected by PV can also develop flaccid blisters on the skin and cutaneous erosions (7), which are not detected in OLP patients (2). Furthermore, esophageal involvement in OLP is usually asymptomatic and can be incidentally detected by esophagogastroduodenoscopy (2), while PV patients describe odynophagia in case of esophageal erosions (8). PV is serologically characterized by the presence of IgG autoantibodies directed against desmoglein (Dsg) 3 and, in case of involvement of the skin, also against Dsg1 (7). These autoantibodies can be detected in serum of PV patients by enzyme-linked immunosorbent assay (ELISA) and also in tissue by direct immunofluorescence (DIF) (9). Dsg, which are variable responsible of the cell-cell interaction in the skin and oral mucosa, show four cadherin repeats in their extracellular (EC) domains and a membrane-proximal extracellular anchor domain (7). The amino-terminal EC1 and EC2 domains are usually targeted by PV autoantibodies, which are necessary and sufficient to cause PV (9).

Rarely, both anti-Dgs1 and anti-Dgs3 autoantibodies have been detected in patients with erosive OLP (10, 11). However, their possible role in the pathogenesis of OLP still needs to be elucidated. In this paper, we reviewed the current literature on the detection of anti-Dsg autoantibodies in OLP, summarizing the most important aspects known so far.

## Discussion

In 2006, the group of Kusic analysed a cohort of 57 patients with OLP (11). The diagnosis was confirmed by histology, and other erosive dermatoses of the oral mucosa were ruled out by DIF. Using ELISA, the authors found out in this retrospective study that the level of both anti-Dsg1 and anti-Dsg3 IgG antibodies was significantly higher in patients with erosive OLP compared to healthy control (HC), patients with recurrent aphthous ulceration, and patients with non-erosive OLP (11). Furthermore, these findings were confirmed by indirect immunofluorescence (IIF) (11). The authors concluded that anti-Dsg IgG autoantibodies could play a role in the pathogenesis of erosive OLP, which could be different to the pathogenesis of non-erosive OLP (11). However, they could not demonstrate the pathogenicity of these autoantibodies and the anti-Dsg1 and 3 autoantibodies values in all patients were below the cut-off level of the ELISA (11).

Several years later, a retrospective study on a cohort of 22 patients with OLP was conducted (12). In this cohort, 15 patients suffered by erosive OLP (12). Using a commercial ELISA (MBL International Corp.), they detected IgG autoantibodies against Dsg3 in a 61-year-old woman with erosive OLP (12). However, none of the 22 patients showed anti-Dsg1 autoantibodies (12). The clinical diagnosis of erosive OLP was confirmed histologically and the DIF did not detect the typical honeycomb IgG pattern of PV (12). In addition, a paraneoplastic pemphigus was ruled out based on the longstanding character of the mucosal erosions, the lack of consistent immune depositions by DIF, the negativity of the IIF on rat bladder, and a negative screening for neoplasia (12). Interestingly, levels of anti-Dsg3 antibodies were related to the clinical activity of the disease, that was evaluated using the Autoimmune Bullous Skin Disorder Intensity Score (ABSIS) (13). Because of the refractory course of the disease, the patient was treated with topical fluocinolone 0.05%, oral methylprednisolone, and mycophenolic acid (12). The authors concluded that the production of anti-Dsg3 IgG autoantibodies was probably stimulated by the exposition of intercellular proteins due to the intense and chronic inflammation (12).

In 2014, the case of a 43-year-old man with a six-month history of refractory oral erosions was described (14). By ELISA, IgG autoantibodies against both Dsg1 and 3 were detected (14). The diagnosis of erosive OLP was confirmed by histology and a PV was ruled out because of the negative findings by DIF and IIF (14). In this case, an improvement of the oral lesions was reported after a therapy with topical tacrolimus (14). Furthermore, the Dsg1 and 3 levels were not correlated to the clinical findings (14). Indeed, IgG autoantibodies against Dgs1 and 3 were detected also after the complete clinical remission (14). Also in this case report, the authors concluded that the severe damage of keratinocytes could induce the production of anti-Dsg antibodies (14).

Two years later, two patients with erosive OLP with high levels of anti-Dsg1 and 3 autoantibodies were described in a case report (15). Actually, one patient, a 68-year-old Japanese woman with a three-month history of painful oral ulceration, showed both anti-Dsg 1 and 3 IgG autoantibodies by ELISA, while the other one, an 85-year-old Japanese woman with a 15-year history of painful oral ulcerations, showed only IgG autoantibodies against Dsg3 by ELISA (15). The diagnosis of erosive OLP was confirmed by histology and by the absence of positive findings by DIF and IIF (15). To better characterise the role of the anti-Dsg3 antibodies, the authors performed ELISA with or and without pre-treatment of the substrate with ethylenediaminetetraacetic acid (EDTA) (15). The ELISA index values for IgG autoantibodies against Dsg3 were not markedly different between EDTA-treated ELISA and untreated ELISA, suggesting that the autoantibodies against Dsg3 were Ca2+-independent and non-pathogenic in both cases (15). Furthermore, the authors performed an ELISA with the precursor and the mature forms of Dsg3 as substrates, showing that the autoantibodies targeted epitopes of prosequence-possessing Dsg3, which explained partially why *in vivo* deposition was not detected by IIF and DIF (15). Both patients were treated with topical tacrolimus and showed an improvement of the oral lesions, but a correlation between the clinical features and the serological levels of anti-Dsg1 and 3 autoantibodies was not evaluated (15).

In 2017, a retrospective study was conducted on a cohort of 113 individuals, including 24 patients with erosive OLP, 29 with non-erosive OLP, 30 patients with cutaneous lichen planus (CLP), and 30 HC (16). The diagnosis was based on clinical features and was confirmed by histology. Levels of circulating autoantibodies against Dsg1 and 3 were determined by ELISA on serum samples (EUROIMMUN, Medizinische Labordingnostika AG, Lubeck, Germany) (16). Furthermore, the activity of OLP was evaluated using Reticulation, Erosion and Ulceration (REU) scoring system (17). Although in all cases serum levels of anti-Dsg3 antibodies were in the normal range, a difference was detected between the level of anti-Dsg3 in erosive OLP and HC (*p* value=0.005) using the Mann–Whitney test (16). Regarding the serological concentration of anti-Dgs1, no difference between the four groups (*p* value = 0.748) was found using the Kruskal–Wallis analysis (16). Furthermore, no correlation was detected between clinical activity and anti-Dsg1 and 3 serum levels in patients OLP (16). Based on these findings, the authors concluded that serum levels of anti-Dsg3 antibodies in patients with erosive OLP were significantly increased in comparison with HC, although the serum levels of the anti-Dsg3 autoantibodies were under cut-off values (16).

A cross−sectional epidemiological study on the prevalence of autoantibodies in patients with several forms of lichen planus was conducted in Mumbai (10). In this study, 100 patients with CLP and OLP were tested for several circulating autoantibodies by IIF, including anti−Dsg1 and 3 too (10). In this cohort, 13 patients had OLP and ten were affected by both CLP and OLP, and nine out of these ten patients showed erosions of the oral mucosa (10). Anti−Dsg1 and 3 autoantibodies were reported respectively in 19% and 16% of the whole cohort of 100 patients, without specifying which patients showed these antibodies (10). Therefore, this study did not help to better understand the possible role of anti-Dsg1 and 3 autoantibodies in OLP.

Also our group evaluated the possible role of autoimmunity in the pathogenesis of OLP (18). Indeed, we reported that patients with OLP and with both CLP and OLP showed an increased peripheral blood Th1-dominated cell response against Dsg3 by ELISpot assay (18). Moreover, we showed in this perspective study that both OLP and CLP are characterized by a peripheral blood Th1/Th17-dominated cell response, which identifies the Dsg3 as target of the inflammation (18).

Because of lack of more detailed studies and due to the limited number of OLP patients with serological evidence of IgG autoantibodies against Dsg1 and 3, a clear role of these autoantibodies in the pathogenesis of OLP cannot be confirmed. Indeed, only in one paper the potential pathogenicity of IgG autoantibodies against Dsg3 was evaluated, showing that they were not pathogenetic (15).

Regarding the production of anti-Dsg1 and 3 autoantibodies in OLP, it seems possible that the humoral epitope spreading (ES) plays a pivotal role. ES consists in the diversification of B and/or T-cell response from an initial dominant epitope to a secondary epitope over the time (19). Two different subtypes of humoral ES have been described, namely the intramolecular and the intermolecular ES (19). In the first case, the diversification of immune response occurs in the same autoantigen, while the intermolecular ES involves different antigens of a single complex or that co-localize in the same anatomical site (19). Because it has been widely reported that chronic inflammation (as in erosive OLP) can lead to ES and several cases of ES have been described in autoimmune blistering diseases (e.g. PV and bullous pemphigoid), it is reasonable that humoral ES can lead to the production of IgG autoantibodies against Dsg1 and 3 in patients affected by erosive OLP (19).

## Conclusion

The detection of IgG antibodies against Dsg1 and 3 is extremely useful to confirm the diagnosis of autoimmune bullous diseases of the pemphigus group (7). Noteworthy, these autoantibodies have been reported also in patient with OLP (10–12, 14–16). At present, the pathogenicity of IgG autoantibodies against Dsg1 and 3 in OLP has been neither in animal models nor *in vitro* evaluated. Indeed, the scientific literature on this topic is limited to case reports and retrospective studies. Further studies are needed to better clarify this point, especially in view of the potential role of detection of IgG antibodies against Dsg1 and 3 for the diagnose and treatment of OLP. Indeed, the presence of these autoantibodies in OLP may be linked to a refractory disease that could be treated with systemic therapies.

## Author contributions

Concept and writing: DD. Revision and editing: MH. All authors contributed to the article and approved the submitted version.

## Funding

This paper was supported by grants from FOR 2497 Pegasus to MH.

## Conflict of interest

The authors declare that the research was conducted in the absence of any commercial or financial relationships that could be construed as a potential conflict of interest.

## Publisher’s note

All claims expressed in this article are solely those of the authors and do not necessarily represent those of their affiliated organizations, or those of the publisher, the editors and the reviewers. Any product that may be evaluated in this article, or claim that may be made by its manufacturer, is not guaranteed or endorsed by the publisher.
